# Does entrepreneurship ecosystem influence business re-entries after failure?

**DOI:** 10.1007/s11365-020-00694-7

**Published:** 2020-08-29

**Authors:** Maribel Guerrero, Jorge Espinoza-Benavides

**Affiliations:** 1 Faculty of Economics and Business, Universidad del Desarrollo, Av. Plaza 680, San Carlos de Apoquindo, Las Condes, Santiago de Chile, Chile; 2 The Northumbria Centre for Innovation, Regional Transformation and Entrepreneurship (iNCITE), Newcastle University Business School, Northumbria University, Sutherland Building 2 Ellison Pl, Newcastle upon Tyne, United Kingdom; 3 Facultad de Ciencias Económicas y Administrativas, Universidad Católica de la Santísima Concepción, Alonso de Ribera, 2850 Concepción, Chile

**Keywords:** Human capital, Social capital, Institutional theory, Entrepreneurial ecosystems, Re-entrepreneurship, Business failure

## Abstract

Previous studies have found a close relationship between exit/failure decisions and entrepreneurial/organisational characteristics. In the same line, entrepreneurship literature has recognised that the context matters in any entrepreneurial process, including “exit,” “failure” or “re-entry.” This manuscript proposes a conceptual framework to identify the elements of the entrepreneurial ecosystem that foster or impede the re-entry into entrepreneurship after a business failure. By reviewing the accumulation of knowledge, we identified the individual, the organisational, and the contextual conditions that influence the trajectory of an individual who decides to re-enter after a business failure. This manuscript provides a better understanding of the critical role of agents involved in the entrepreneurial ecosystem. A provocative discussion and implications emerge for this study in order to reduce individual barriers and unfavourable social norms towards business failure.

## Introduction

The entrepreneurial process consists of several stages that are configured through the combination of a series of individual, organisational, and contextual factors (DeTienne, 2010; Shepherd et al., 2019). In the last years, due to the positive effect of entrepreneurship on economic growth, academia and policymakers have paid their interest on the entrepreneurial ecosystem’s pillars (Stam, 2015; WEF, 2014). Previous studies have explored some contextual conditions that foster high-growth based entrepreneurship^1^ (Acs et al., 2017a and b; Brown & Mason, 2017; Mason & Brown, 2013, 2014). Notably, the Silicon Valley model of entrepreneurship has captured not only the imagination of the public, but also the attention of the public policy community throughout the world who wish to emulate said model, and the focus of scholars seeking to understand it (Audretsch, 2019). However, this model presents several limits when addressing the most compelling contemporary economic and social problems across the globe.

By looking into the existing literature, it is possible to identify the particular conditions that act as drivers or barriers for entrepreneurship across the globe (Guerrero et al., 2020). Previous exit/failure studies have recognised the significant influence of entrepreneurs and organisational characteristics on the exit/re-entry decisions (Sheppard and Chowdhury, 2005; Ucbasaran et al., 2009, 2010, 2013). Industrial organisation and organisational ecology studies have also highlighted the relevance of contextual conditions on business failure (Mellahi & Wilkinson, 2004). Although the influence of the context across the entrepreneurship process has been studied, the influence on entrepreneurship exit/re-entry has been unexplored (Cardon et al., 2011; Khelil, 2016; Raffiee & Feng, 2014; Walsh & Cunningham, 2016). More concretely, the role of the entrepreneurial ecosystem (or context) on re-entry decisions after a business failure (Hsu et al., 2017; Simmons et al., 2018; Ucbasaran et al., 2013). The context could affect, for example, psychological and emotional recovery processes from business failure, and the speed/quality of business re-entry after failure(Corner et al., 2017; Guerrero & Peña-Legazkue, 2019; Williams et al., 2019).

Based on an exhaustive review of the accumulation of knowledge about failure, re-entry, and entrepreneurial ecosystem (Rauch, 2019), this paper proposes a conceptual framework to identify the entrepreneurial ecosystem elements that foster or impede the re-entry into entrepreneurship after a business failure. Our study contributes to two academic debates in entrepreneurship: (a) the role of ecosystems across the entrepreneurship process (Audretsch, 2019), and (b) the individual, organisational and contextual determinants of new re-entries after business failures (Walsh, 2017).

After this introduction, this paper is structured as follows. Section 2 describes the literature review of the factors that have determined the exit decision, re-entry, and those that make up the entrepreneurial ecosystem. Section 3 presents the literature review that exposes the pillars of the entrepreneurial ecosystem and the specific conditions that may influence the re-entry process after an entrepreneurial failure. Section 4 discusses the proposed model and contributions. Section 5 describes the main conclusions, limitations, implications, and future lines of research.

## Theoretical foundations

### Business failure/exit

Business “exit” or “failure” has been analysed from different perspectives: economic-financial, accounting, legal, strategic, organisational, and business. The interpretation of this phenomenon depends on the adopted theoretical approach. Hessels et al. (2011, p. 450) describe business exit as the permanent closure, sale, discontinuance, or abandonment of a business. Complementary to this, Ucbasaran et al. (2013, p. 175) describe business failure as the cessation of involvement in business because of the lack of achievement of the minimum economic expectations stipulated by the entrepreneur. Both definitions are related to the cessation of an entrepreneurial initiative derived from individual decisions, organisational characteristics, and contextual conditions. From business success/failure perspectives, different internal and external elements conditioned the occurrence of both events across the entrepreneurial process (Zacharakis & Meyer, 1999; Sheppard and Chowdhury, 2005). Table 1 shows the internal and external determinants of business failure.

**Table 1:** Determinants of business exits/failures

**Determinants**	
**Internal**	**External**
**Entrepreneur** (Hayward et al., 2010; Hessels et al., 2011; Khelil, 2016; Ucbasaran et al., 2009, 2010, 2013; Walsh & Cunningham, 2016) • Decisions and actions that are under control • Human capital: lack of knowledge, lack of skills, lack of abilities, lack of previous managerial or entrepreneurial experiences • Personal characteristics: lack of confidence, risk-aversion **Organisation** (Gaskill et al., 1993; Khelil, 2016) • Lack of financial planning • Lack of investment capital or liquidity • Lack of social capital • Lack of organisational capacity	**Context ** (Ucbasaran et al., 2009, 2010, 2013; Cardon et al., 2011; Khelil, 2016; Stephen & Wilton, 2006; Vaillant & Lafuente, 2007) • Events beyond the control of the entrepreneur • Social, economic, political, natural circumstances of the country • Fiscal policies • Labour policies • Financial policies and support related to access to credit or loans • Quality of institutions • Culture

Source: Authors

The majority of studies have focused on individual and organisational factors as the crucial determinants of a business exit/failure decision (Ucbasaran et al., 2009, 2010, 2013). Individual characteristics (age, education, experience, the propensity to risk, confidence, resources, capabilities) shape entrepreneurs’ decisions (Cardon et al., 2011; Walsh & Cunningham, 2016). Therefore, the lack of skills and the lack of liquidity have been the leading causes of business failure or exit (Gaskill et al., 1993; Hayward et al., 2010; Hessels et al., 2011; Walsh, 2017; Walsh & Cunningham, 2016). Although the academic debate primarily focused on internal and organisational factors (Cardon et al., 2011; Gaskill et al., 1993; Liao et al., 2008), a few studies have associated business failure with external conditions as the level of unemployment, tax, per capita income, percentage of business entries/exits, government changes, technology, and market conditions. Previous studies have also identified that the lack of regulatory, fiscal, and financial frameworks that support business creation and development (Stephen & Wilton, 2006), as well as the poor quality of institutions (Vaillant & Lafuente, 2007), have been associated with failure and exit. In this vein, Sheppard & Chowdhury (2005) identified the critical role of organisational interactions and managers’ strategic adjustments on business failure instead of contextual conditions.

### Re-entrepreneurship after a business exit/ business failure

The entrepreneurial process presents events and interactions between the entrepreneur, the organisation, and the context in a determined space and time. According to Kang & Uhlenbruck (2006), entrepreneurial actions depend on cyclical and dynamic processes of exploration and exploitation of business opportunities. Consequently, entrepreneurs decide the entrepreneurial trajectory of their initiatives: the continuity, the exit, or the re-entry. In general, the entrepreneurial process begins with an exploration of opportunities (discovering, searching, selecting) that can move towards the exploitation (organisation, negotiation, strategy, and learning) and then to a potential survival (re-investment, strategical orientations, growth), decline (liquidation, de-investment) or re-entry into the process (Kang & Uhlenbruck, 2006, p. 49). Along the entrepreneurial process, entrepreneurs may move from exploration to exit without going through exploitation or even move from exploration to re-entry without going through an exit (DeTienne, 2010; Shepherd et al., 2019). Consequently, an exit or business failure will modify individuals’ motivations. Some entrepreneurs would focus on stable employment alternatives, while other entrepreneurs would assume higher risks looking for self-employment alternatives like becoming investors or re-entering the entrepreneurial process (Parker & Van Praag, 2012; Kang & Uhlenbruck, 2006; Ucbasaran et al., 2006, 2013; Parker, 2013; Burton et al., 2016). Both cases have provided insights into the positive and negative effects of business failures (Table 2).

**Table 2:** Effects of business exits/failures

**Effects**	
**Positive**	**Negative**
**Entrepreneur** (Atsan, 2016; Cope, 2003, 2011; Khelil, 2016) • Experience to access information linked to previous business activity that reduces the opportunity cost • Experience to explore and exploit opportunities • Business management experience • Building networks and contacts **Organisation** (Cope, 2011; Jenkins et al., 2014; Khelil, 2016) • Understanding how to improve financial indicators **Context ** (Parker, 2013) • Encourage the development of favourable policies towards entrepreneurship re-entry	**Entrepreneur** (Cardon et al., 2011; Cope et al., 2004; Simmons et al., 2014) • Lack of confidence and optimism • Fear of failure • Assuming lower risks/business projects due to assumed costs **Organisation** • …. **Context ** (Cardon et al., 2011; Kerr & Nanda, 2009; Haselmann & Wachtel, 2010; Simmons et al., 2014) • The negative perception of business failure in society • The lack of regulatory frameworks to access to credits

Source: Authors

Regarding the positive effects of business failure, previous studies have shown favourable effects of business failure on entrepreneurs. First, business failure helps to identify personal strengths and weaknesses (i.e., skills, attitudes, knowledge, and beliefs) that are very useful across the entrepreneurial process (Jenkins et al., 2014). Second, business failure represents an opportunity to identify organisational strengths and weaknesses (i.e., customer information, market, liquidity, production, and innovation) that are useful in the exploration of business opportunities and the reduction of exploitation costs (Atsan, 2016). Third, business failure contributes to building strategic networks and social relationships that may be transformed into dynamic capabilities in future ventures (Cope, 2011). Four, previous business experiences reveal the need for leadership and managerial roles as well as the notion of high-level learning due to the occurrence of discontinuous events of small organisations (Cope, 2003).^2^ Fifth, the serial entrepreneurship literature has evidenced higher (but temporal) economic-financial benefits due to the previous failure learning processes as well as the spillover effects (Parker, 2013; Khelil, 2016). In this respect, Parker (2013) highlighted the importance of public policies that promote/strengthen the re-entry into entrepreneurship even if they generate performance indicators lower than their previous companies. Public policies oriented to support re-entries after failure should consider the entrepreneur’s experiences and trajectories (Corner et al., 2017). It implies that not all re-entrepreneurs demand public/private support to face failure (Williams et al., 2019).

Regarding the adverse effects of business failure, previous studies have identified four adverse effects of business failure on entrepreneurs. First, individuals’ attitudes and behaviours have negatively influenced by the cultural stigma of failure in sanctioned societies (Cardon et al., 2011; Simmons et al., 2014). Second, the socialisation process has negatively influenced individuals’ risk-taking and career decisions as re-starting a venture or seeking paid work (Cope et al., 2004). Third, the adverse effects on specific procedures or regulations associated with the restricted access to credits or grants after a business failure (Kerr & Nanda, 2009; Haselmann & Wachtel, 2010). Fourth, re-entrepreneurs will have to face structural barriers like access to innovation/knowledge, cost disadvantages, capital requirements, government licenses, financial risks, as well as strategic barriers such as strategic behaviours, collusion, information asymmetries, and lack/excess of capacities (Lutz et al., 2010).

### Entrepreneurial ecosystem

The entrepreneurship literature has given the context an indisputably important role in the promotion of entrepreneurial activity as well as in the impact on the economic development of a territory (Hoskisson et al., 2011). According to the institutional theory (North, 1990:3), institutions are “the rules of the game in a society” that can be formal (laws, regulations) and informal (attitudes, values, social norms). By adopting this approach, it is possible to identify formal and informal conditions that have influenced entrepreneurial entries and re-entries. An institutional framework is required to facilitate/promote entrepreneurial culture in a territory as well as interrelations/cooperation between entrepreneurs, organisations and other agents (Brown & Mason, 2017). The called “entrepreneurial ecosystem” (Acs et al., 2017a and b) has emerged based on these relationships. This terminology has been used to understand the interconnected group of entrepreneurs (potential, nascent and existing), financing agents (companies, venture capitalists, business angels, banks), and promoting organisations (universities, public sector agencies) that converge to support entrepreneurial initiatives (social, inclusive, high growth potential, serial) oriented to generate value in the territory (Mason & Brown, 2014, p.5). The analysis of entrepreneurial ecosystems has been crucial to the development of public agendas (Acs et al., 2017a and b). According to Stam (2015, p. 6), an entrepreneurial ecosystem is configured by institutional pillars (formal institutions, culture, physical infrastructure, and demand) and systemic elements (networks, leadership, financing, talent, knowledge, and intermediaries) that support the development of high-growth entrepreneurial initiatives (technology-based entrepreneurship or corporate entrepreneurship) to generate productivity, income, employment and well-being in the region. Table 3 describes the entrepreneurial ecosystem pillars that reinforce the individual and organisational determinants of entrepreneurial initiatives (Herrmann et al., 2012; WEF, 2014; Simón-Moya et al., 2014). Indeed, an entrepreneurial ecosystem is a dynamic and evolutionary process that ensures the creation of high-potential entrepreneurship that generates growth, productivity, and well-being (Stam & Spiger, 2016).

**Table 3:**
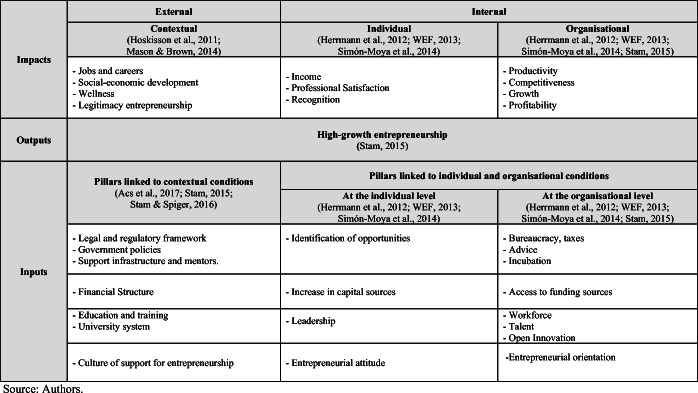
Entrepreneurial ecosystem and its influence on the determinants of entrepreneurial activity

## Linking re-entries after a business failure and entrepreneurship ecosystem

The accumulation of literature on business failure highlighted three premises. First, failure can be caused by shortcomings/errors linked to the entrepreneur and external conditions that are beyond the control of the entrepreneur. Second, business failure can generate some favourable and adverse effects that determine subsequent labour decisions. Three, even though entrepreneurial ecosystems are oriented towards high growth entrepreneurs, the ecosystems’ pillars, directly and indirectly, contribute to re-entry processes by reducing the adverse effects or weaknesses after a business failure. Based on these premises, Table 4 shows the theoretical framework linking the internal and external factors associated with business failure, the favourable and adverse effects faced in re-enterprise processes, as well as the role of the pillars that make up the entrepreneurial ecosystem (Acs et al., 2017a and b; Stam, 2015; Stam and Spiger, 2016). These pillars were adopted to understand the elements that foster re-entrepreneurship after a business failure. We discussed five pillars of the entrepreneurial ecosystem and proposed some propositions related to the process of re-entrepreneurship after a business failure.

**Table 4:** Entrepreneurial ecosystem and re-entrepreneurship

**Entrepreneurial ecosystem pillars**	**- Regulatory frameworks** **- Government policies**	**- Financial Structure**	**- Support infrastructure and Mentors**	**- Education and training** **- University system**	**- Culture of support**
**Re-entrepreneurship (covering or reinforcing weaknesses after business failure)**	- Establishment of policies and programs that encourage re-entrepreneurship (as a mechanism of the legitimacy of business failure) (Kerr &Nanda, 2009; Parker, 2013; Ucbasaran et al., 2003; Walsh, 2016)	- Access to credit or sources of capital by valuing the project and the entrepreneur’s experience rather than hardening the procedure following the failure (Atsan, 2016; Chakrabarty & Bass, 2013; Cope et al., 2004; Kehlil, 2016; Kerr & Nanda, 2009; Parker, 2013)	- Providing advice - Design of workshops in which they participate and disseminate experiences of business failure or re-enterprises to support other entrepreneurs in the system (Cannon & Edmondson, 2001, 2005; Cope, 2011; Walsh, 2016)	- Strengthen personal weaknesses and those linked to entrepreneurial activity - Strengthen processes to raise awareness of business failure -Design of education and training programmes **(**Amaral et al., 2011**;** Hsu et al., 2017; Ucbasaran et al., 2006, 2009; WEF, 2014)	- Dissemination of experiences of business failure and re-entrepreneurship - Sensitise society to failure as a process of learning and growth instead of punishing it with the belief that it is something negative. (Atsan, 2016; Cardon et al., 2011; Khelil, 2016; Ravindran & Baral, 2014; Shepherd & Wiklund, 2006; Stuetzer et al., 2018)

Source: Authors

### Regulatory framework and government programmes

An entrepreneurial ecosystem has an appropriate regulatory framework for strengthening high-growth entrepreneurs. The regulatory framework represents governmental policies and programmes oriented to reinforce innovative collaborations and technology transfer processes and to foster the development of high-growth entrepreneurship that generates societal value and spillover effects (Stam, 2015). Regarding re-entrepreneurship after a business failure, regulatory frameworks should be oriented to reduce the stigma of failure (Walsh, 2016). Extant investigations have identified public policy initiatives oriented to provide adequate fiscal, financial, and legal conditions for re-entrepreneurs (Gentry & Hubbard, 2000; Cuthbertson & Hudson, 1996; Kerr & Nanda, 2009; Parker, 2013; Walsh, 2016). Regarding taxes, Gentry & Hubbard (2000) found that higher levels of taxes affect the entrepreneurs’ entries or re-entries, as well as the significant relationship between higher taxes and business failure. Therefore, if the lack of fiscal incentives explains adverse effects on entrepreneurship, regulatory frameworks should understand this negative effect on business entry/exit and include efficient regulations to promote saving and investments. Regarding financial regulations, Kerr & Nanda (2009) and Parker (2013) emphasizes on the revaluation of practices of financial organisations. These organisations must give more value to the track record of the re-entrepreneur independently if he/she changed the industrial sector in the new entrepreneurial initiative (Kerr & Nanda, 2009). Also, these organisations must change bankruptcy laws regarding the depreciation of the financial trajectory in cases of re-entrepreneurship (Cuthbertson & Hudson, 1996). Regarding legal conditions, the design and implementation to support re-entrepreneurship should also be as part of the regulatory framework within an entrepreneurial ecosystem (Hoskisson et al., 2011; Ucbasaran et al., 2003). Based on these assumptions, we propose the following proposition:

#### Proposition 1:

- *Entrepreneurial ecosystems characterised by public policies and programs that have been designed to minimise the costs of business failure will provide favourable conditions for new re-entries into entrepreneurship*

### Access to financing sources

Access to sources of finance or capital is usually one of the main barriers for entrepreneurs at each stage of their entrepreneurial initiative (Chakrabarty & Bass, 2013; Kehlil, 2016). Therefore, in re-entry into entrepreneurship, this entrepreneurial ecosystems pillar is crucial to create a new business as well as to reduce the social stigma of failure. Previous experiences about (un)successful economic-financial management reinforce the ability to anticipate difficulties during the creation of new business in similar or different sectors (Atsan, 2016; Kerr & Nanda, 2009). Consequently, the experience gained from business failure should be an advantage instead of a disadvantage when entrepreneurs are applying for a loan or presenting the project to investors (Atsan, 2016). Previous business failure experiences represent knowledge acquired regarding accounting, economic, financial, strategic management, and social networks, and thus, the opportunity to reduce the social stigmatisation of business failure (Stam, Audretsch, & Meijaard, 2008). In the European context, investing/granting in entrepreneurial activity from individuals who have experienced business failures represents a high risk of return on investment or credit (Cuthbertson & Hudson, 1996; Zacharakis et al. 1999). Directly or indirectly, due to the stigmatisation of failure, the perception of European investors represents a barrier for entrepreneurs when the investors’ decision should be based on the proposal: quality, feasibility, expectations and limitations/risks (Cope et al. 2004). At the financial pillar, it is crucial to modify the stigma of considering business failure has a negative mark for the entrepreneur when they are presenting a new proposal to the business capital community or in the financial market (Parker, 2013). From the legal perspective, studies on corporate liquidation have found legal loopholes as well as the lack of legitimacy during the liquidation of a company (Cuthbertson & Hudson, 1996). Business failure or exit should be seen as a natural stage of any dynamic and entrepreneurial process. Based on these arguments, we propose the following proposition:

#### Proposition 2:

- * Entrepreneurial ecosystems characterised by a financial system/investors that have positively evaluated the business failure experience will provide favourable conditions for new re-entries into entrepreneurship*

### Physical infrastructure, professional and mentoring support

Physical and professional infrastructures (incubators/accelerators) are critical pillars within the entrepreneurial ecosystem (Stam, 2015). These infrastructures have mentors who provide their services in strategic areas (strategic, legal, accounting, financial, marketing, innovation, and production) for re-entrepreneurs. An example of good practices has been the participation of entrepreneurs who have experienced business failures through talks, networks or workshops as part of the advisory work team.^3^ Cope (2011) considers that this type of actions facilitates the learning process after a business failure based on feedback and collaborative reflection about business, social, emotional and intellectual aspects among the participants. In this respect, Cannon & Edmondson (2001, 2005) also found that working in groups mitigates the negative perception of business failure because people transform into personalised mentors who transfer experience and knowledge to those who are facing critical events. In this vein, Walsh (2016) suggested the formalisation of professional assistance and mentoring for re-entrepreneurs by providing access to networks, resources, and support structure. Corner et al. (2017) also suggested that there are types of resilient entrepreneurs who do not require a process of recovery and support, as they quickly move on from the experience of failure to re-start a business. In this regard, Williams et al. (2019) found that these types of entrepreneurs may have learned very little from the experience of failure and, therefore, replicate some decisions/behaviors that increase the likelihood of further failure. Based on these arguments, we propose the following proposition:

#### Proposition 3:

- *Entrepreneurial ecosystems characterised by support infrastructures that have included mentors with previous business failure experiences will provide favourable conditions for new re-entries into entrepreneurship*

### Education and training programmes

Human capital (experiences, skills, education) is a relevant determinant of business failure/success associated with the education pillar of the entrepreneurship ecosystem (Guerrero & Peña-Legazkue, 2019). The learning process after business failure represents the opportunity to transform the skills, experiences, and knowledge that should be required in new business re-entries. Previous studies have found a negative perception due to the social stigmatisation of failure (Stam et al., 2008). In this vein, Amaral et al. (2011) conducted a longitudinal study of serial entrepreneurs, and they found differences between general human capital (education and previous work experience) and specific human capital (entrepreneurial experience, the management, or investment). Amaral et al. (2011) found that generic human capital slows down the decision to re-enter. In this scenario, previous business failure experience plays a significant role in deciding to re-enter due to the opportunity cost of choosing another occupation (Walsh, 2017). Therefore, the pillar of higher education of an entrepreneurial ecosystem may contribute to reinforcing re-entry decisions (WEF, 2014).

Training and entrepreneurship capacity building programmes reinforced the weaknesses identified in the learning process and linked them with the exploration and exploitation of entrepreneurial opportunities (Ucbasaran et al., 2006, 2009). Similarly, Cope (2003) points out that discontinuous events (i.e., business failure) represented high-level entrepreneurial learnings in terms of understanding rules, defining new actions, and implementing new changes. Previous experiments have also found that the re-entry intention is experiential on entrepreneurs with a moderated self-confidence (Hsu et al., 2017). It implies that the design of training programs or training in entrepreneurship could include/reinforce strategic areas. For example, the analysis of business success/failure cases and the transference of business failure knowledge from higher-level learners to new learners. Based on these arguments, we propose the following proposition:

#### Proposition 4:

- *Entrepreneurial ecosystems characterised by a higher education system that has enhanced specific human capital* via *training entrepreneurship programs will provide favourable conditions for new re-entries into entrepreneurship*

### Entrepreneurial culture

Previous studies suggest that regions with the highest levels of entrepreneurial culture tend to be the highest growth-oriented regions (Stuetzer et al., 2018). The social context has a positive/negative influence on the entrepreneurship phenomenon. Generally, after a business failure, the individual tried to interpret/understand the causes (Shepherd & Wiklund, 2006). In this questioning process, the individual had a sense of loss, low self-confidence, and absence of an optimistic future (Atsan, 2016; Khelil, 2016). In this regard, the individual’s social context could reinforce these attitudes or behaviours (Yamakawa et al., 2015). The social perception of failure must be understood as a learning/experience rather than a sanction (Cardon et al., 2011). Given the nature of cultural factors across generations, it takes time to transform the social sanction of business failure towards a more conducive entrepreneurship culture. Social media, education, and entrepreneurship programs play a crucial role in the stigmatisation of business failure. A strategy could be focusing on the economic, societal, and regional contributions of entrepreneurs who re-entry into the market with new entrepreneurial initiatives after a business failure (Ravindran & Baral, 2014). Based on these arguments, we propose the following proposition:

#### Proposition 5:

- *Entrepreneurial ecosystems characterised by societies that have not penalised the business failure will provide favourable conditions for new re-entries into entrepreneurship*

## Proposed conceptual framework

**Fig. 1:**
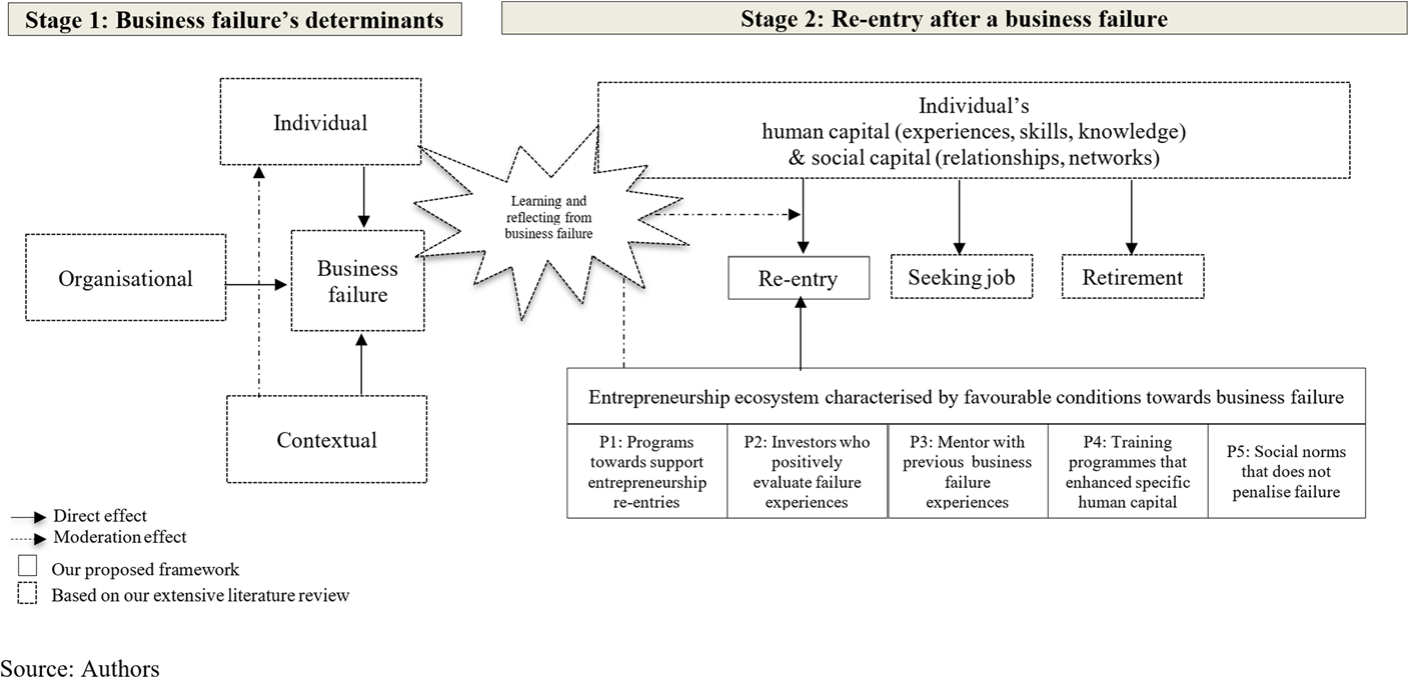
Conceptual framework

Based on our literature review, Fig. 1 shows the two traditional determinants of business failure (individual and organisational), as well as the individual’s potential decisions after a business failure (re-entry into entrepreneurship, seeking a job or retirement).

Our contribution focused on the academic discussion about the influence of contextual conditions in the entrepreneurs’ trajectory (Acs et al., 2017a and b; Audretsch et al., 2019). Our model proposes an overall better framework to look at the re-entry entrepreneurship phenomenon by introducing the role of the entrepreneurship ecosystem (see Table 3 and Table 4). Primarily, we assumed that an individual had experienced critical reflection and learning from a business failure (Cope, 2003, 2011). Secondly, we assumed that the individual is part of an entrepreneurship ecosystem that provides favourable conditions along the entrepreneurial process. In this assumption, the individual had the opportunity to learn from failure and reinforce specific human capital by the influence of several entrepreneurship ecosystem elements (Simmons et al., 2014). Thirdly, the individual’s decision to participate in a new re-entry into entrepreneurship will be positively related to the existence of support programs (P1), the positive evaluation of business failure experience by the financial system (P2), the existence of mentoring programmes provided by individuals with business failure experiences (P3), the existence of training programmes that reinforced the individual’s human capital (P4), and the existence of social norms that do not penalise business failure (P5). Fourthly, the entrepreneurship ecosystem conditions have a direct influence on individual-specific human capital, as well as a moderation effect on the trajectory of individuals’ entrepreneurial actions (Cardon et al., 2011; Walsh, 2017). Consequently, the individual will be more likely to develop an entrepreneurial initiative with high-growth and innovative orientations (Audretsch, 2012; Sheppard & Chowdhury, 2005).

## Conclusions

The social, economic, health, and political scenarios considerably delimitate the actions of the different agents that make up the entrepreneurial ecosystem. This paper presents the accumulation of knowledge about the individual, organisational and contextual conditions that determined entrepreneurial success, failure, or re-entry (Rauch, 2019). Specifically, our proposed conceptual model contributes to the entrepreneurship literature by highlighting the debate about the role of specific entrepreneurial ecosystem conditions that positively influence re-entry into entrepreneurship after a business failure. Our theoretical framework also extended the discussion about the contextual conditions (Welter et al., 2019; Baker & Welter, 2020) and the dynamism of the entrepreneurship process (Guerrero et al., 2020).

This study has several limitations that should be considered in the re-entry into the entrepreneurship research agenda. First, conceptually, our model proposed five entrepreneurial ecosystem conditions that need to be reinforced by the adoption of complementary theoretical approaches at the contextual level (the institutional economic theory, the evolutionary and the stakeholders’ approach), the individual level (learning, behavioural, psychological and decision-making approaches), and the organisational level (organisational learning, ambidexterity, and dynamic capability approaches). Special attention should be also paid to the re-entrepreneurs’ response to external shakeouts (COVID-19 pandemic, economic crisis/recessions). Second, empirically, the propositions need to be tested through qualitative (case studies, narratives, action-research, experiments) and quantitative (cross-sectional, longitudinal, multilevel) investigations across regions and countries around the world. Diversity should be crucial to understanding the intensity of each entrepreneurship’s ecosystem conditions across different research settings, different cultural backgrounds, different industries, and different types of entrepreneurs’ re-entries. It also demands the implementation of different measures of re-entry into entrepreneurship and ecosystems’ conditions (Iversen et al., 2007; Audretsch, 2019; Dencker et al., 2019; Henrekson & Sanandaji, 2019). The exploration of direct, indirect, moderation and mediation effects of the entrepreneurship ecosystem on re-entry into entrepreneurship should also be considered in future research. The lack of research about how the entrepreneurial ecosystem pillars could consider the importance of the industry in which a venture belongs. Third, the research agenda should also consider the static and dynamic perspectives of both entrepreneurship and ecosystems. The trajectory of entrepreneurship and the evolution of ecosystems are related to speed (Dencker et al. 2019; Henrekson & Sanandaji, 2019) and quality (Guerrero & Peña-Legazkue, 2019) of new entrepreneurship re-entries.

Several implications also emerge from this study. *For potential re-entrepreneurs*, the study offers the identification of the antecedents and the consequences of business failure. Based on the experience of individuals who decided to re-enter into entrepreneurship, it is possible to provide a better understanding of the entrepreneurial trajectories after failure, as well as the role of the entrepreneurial ecosystem in this process. *For policymakers*, the re-entry into entrepreneurship after failure is a phenomenon related to the highest socio-economic costs and benefits. Policymakers should understand re-entrepreneurship after a business failure. We assume that re-entrepreneurs are resilient but may require mentoring support across the re-entry process, as well as psychological to support to overcome traumas produced by failure.

By introducing the notion of the entrepreneurship ecosystem, the accumulation of knowledge about business failure could be disseminated among public agencies, intermediaries, investors, universities, entrepreneurs, and other organisations. This type of dissemination allows a better understanding and legitimisation of business failure. Consequently, it is useful for the implementation of support mechanisms to minimise costs and maximise benefits from re-entries into entrepreneurship.

## Footnotes

1 According to the OECD, high-growth ventures have an average annual growth greater than 20% in terms of employment and profitability (Audretsch, 2012).

2 Cope (2003, p. 433) defined high-level learning as the development of complex rules and associations regarding new actions. It implies the development of an understanding of causation, central norms, frames of reference, and change of assumptions.

3 This is a practice used in support programs involving serial entrepreneurs. There is some example of initiatives promoted by Setting up in the U.K., Kauffman Mindset programs in the U.S., Start-up Chile in Chile, and Entrepreneurship reactivation programs in Japan (Yasuda, 2009).
